# Dynamic home range of the grey-sided vole *Craseomys rufocanus*: a pilot study

**DOI:** 10.1186/s40850-024-00209-9

**Published:** 2024-07-15

**Authors:** Karl Frafjord

**Affiliations:** Tromsø Museum, UiT The Arctic University of Norway, P. O. Box 6050, Langnes, Tromsø, 9037 Norway

**Keywords:** Home range size, Reproduction, Sex difference, Variability

## Abstract

**Background:**

Knowledge about home range size is vital to understand social systems and population dynamics of small mammals, as well as dispersal and a species’ landscape use. Home ranges have been mapped for some species of voles (subfamily Microtinae) but remain virtually unknown for many species, including the grey-sided vole *Craseomys rufocanus*.

**Results:**

A small pilot study was carried out in an inland valley of northern Norway, where six adult *C. rufocanus* were radio-tracked with one male and one female in each of the summers 2021–2023. Despite the small sample size, a large variation in home range size was found; males 2 294 − 36 887 m^2^ and females 1 728-7 392 m^2^ (100% MCP). Three of the voles tracked over a prolonged period of time showed a dynamic use and shifting of the range. Home range size and use was mostly related to reproduction. The male with the smallest range had probably not yet become reproductively active, whereas the male with the largest range was searching for females at a time when vole density was very low. The third male reduced his range when the reproductive season ended. For females the most important limitations were food, shelter and dependent young, those with young needed to return frequently and spend more time at the nest site. When the reproductive season ended, one female increased her range, perhaps exploring sites to overwinter.

**Conclusions:**

Home range use in this population appears to be more dynamic than has previously been reported for *C. rufocanus*. The large ranges of males most likely resulted from the search of reproductively active females, outside of the reproductive season male ranges approximated female ranges. Female ranges most likely were limited by the need to feed close to their nest with dependent young, being able to roam more freely when reproduction ended.

**Supplementary Information:**

The online version contains supplementary material available at 10.1186/s40850-024-00209-9.

## Background

The concept of home range is well established for terrestrial mammals, although not always easily interpreted [1, 2]. A home range may or may not be larger than a more actively defended territory, which contains the most important resources such as food, shelter or dens [3, 4]. Most territorial carnivores have reasonably well-defined home ranges, although some are exceptionally large. Herbivores such as ungulates often have less well-established home ranges, especially those that embark on long seasonal migrations. Home range size may vary within a species largely dependent on habitat quality, most importantly food availability, but may also be related to season, population density, reproduction, sex, size or mass [1].

The knowledge of home range size in voles (subfamily Microtinae) is scant and often difficult to compare due to different methods used. Several studies were based on the capture-mark-recapture (CMR) method, by which ranges may be substantially underestimated [5, 6]. Radio-tracking studies are often short-term and based on relatively few positions, and again may underestimate ranges. Most reported home ranges are in the order of 0.03–0.5 ha, equivalent to 300-5 000 m^2^ [7–12], but in some cases larger [13–18]. In the bank vole *Myodes* (formerly *Clethrionomys*) *glareolus*, maximum male and female home range sizes were 11 000 and 4 850 m^2^ respectively (CMR), with males increasing their range in late winter and spring [13, 19]. In an experimental population of this species, home range size of females decreased during the reproductive cycle and did not correlate with female weight [9]. In the breeding season, most female microtine rodents are considered territorial with mostly exclusive home ranges, while males use larger and more overlapping ranges in search of females [5, 6, 9, 11, 15, 16, 20–23]. In winter these voles are generally believed to be less territorial.

The grey-sided vole (or grey red-backed vole) *Craseomys*, formerly *Myodes* [24], *rufocanus* is distributed across the northern part of the Euro-Asiatic continent, from Norway through Siberia to the Pacific coast [25]. Based on CMR, a mean home range size of 1 446 m^2^ in males and 351 m^2^ in females was found [26], as well as 1352 m^2^ in females [27]. Other studies have shown that females have exclusive home ranges, while males usually have larger and overlapping ranges [28, 29]. However, neighbouring females may more frequently come from the same maternal lineage and females may aggregate into kin groups (clusters), in some cases even mixed lineage groups [30, 31]. No radio-tracking studies under fully natural conditions seem to have been performed anywhere, two studies used restricted areas [26, 32].

In this study the main aim was to map the home ranges of both male and female *Craseomys rufocanus* across the summer season by radio-tracking. Although it was only a small pilot study with a sample size too small to statistically test what factors may influence the range, such data are very much needed and should give indications as to which factors can be most important. Another aim was to compare the results of radio-tracking with those of the CMR method used by previous studies.

## Methods

**Fig. 1:**
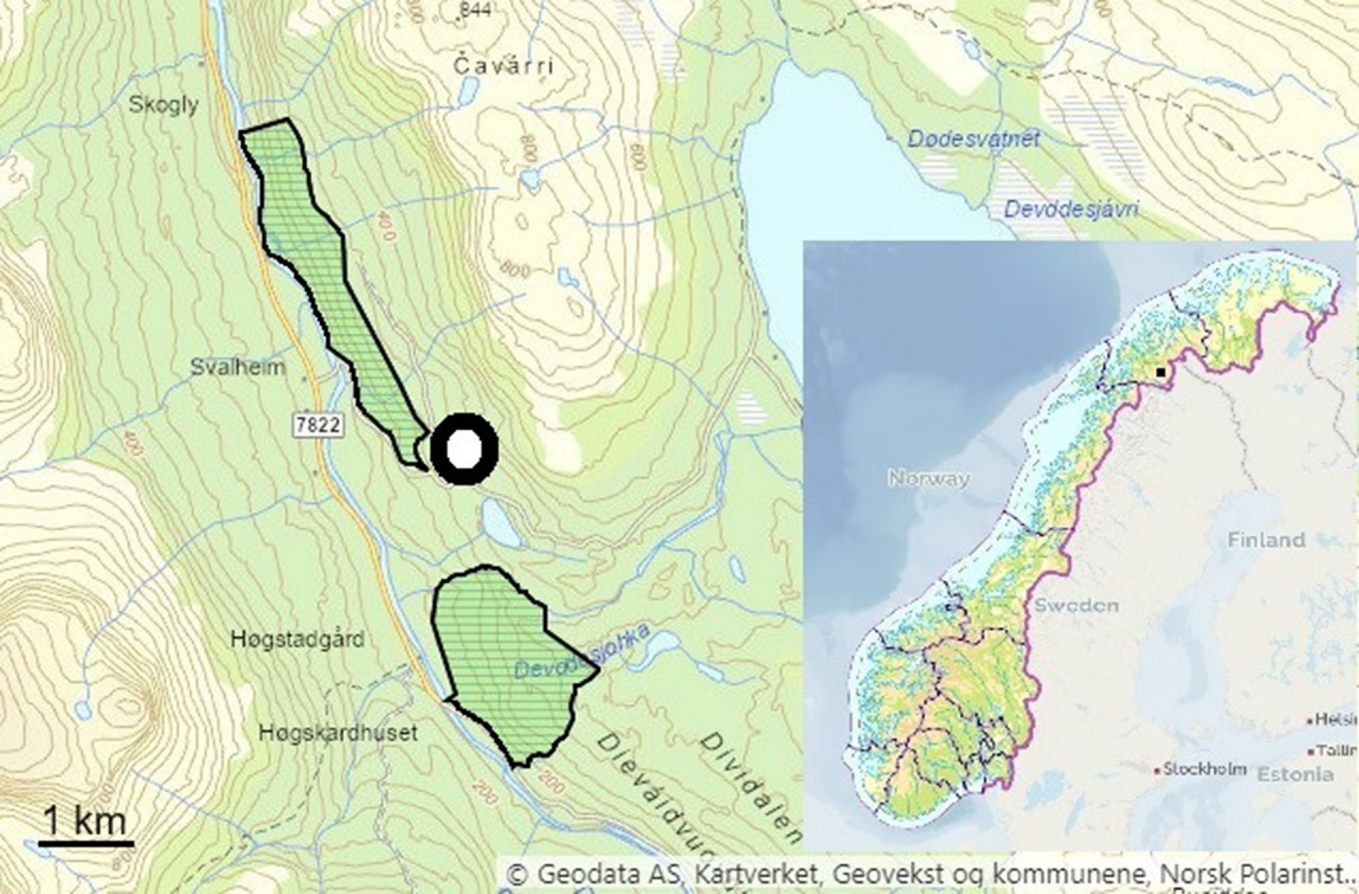
Map showing the location of the study area in Norway (inset, black square) and in the Dividalen valley (black circle). The location of Brennskoglia nature reserve is hatched in green

The study area was 303 m a.s.l. in Dividalen (68° 51’ N, 19° 34’ E), a valley in the eastern part of Troms County, northern Norway (Fig. 1). The habitat in the region is generally crowberry-pine-forest with pine *Pinus sylvestris* and birch *Betula pubescens*. However, few pines were found in the actual study area, which consisted mostly of aspen *Populus tremula* and birch. The ground layer consisted principally of juniper *Juniperus communis*, bilberry *Vaccinium myrtillus*, lingonberry *Vaccinium vitis-idaea* and mountain crowberry *Empetrum hermaphroditum*. Grasses were scarce. Bilberry is a particularly important food for *C. rufocanus* [33]. The forest had not been managed for a long time, never by machinery, included some old and large trees and appeared rather primeval. It bordered a recent forest nature reserve, Brennskoglia naturreservat (Fig. 1), more information about the vegetation in this reserve can be found at: https://faktaark.naturbase.no/?id=VV00003425 (in Norwegian only). Predators recorded infrequently in the study area were stoat *Mustela erminea*, weasel *M. nivalis*, marten *Martes martes*, red fox *Vulpes vulpes*, pygmy owl *Glaucidium passerinum* and hawk owl *Surnia ulula*. Once a family of three brown bears *Ursus arctos* ran through the study area. The climate was typical “inland”, with little precipitation and cold winters. In the years of this study, the snow cover had largely melted by the end of May, when the breeding season of *C. rufocanus* normally commence.

The voles were trapped in GrahnAB’s Ugglan Special small mammal traps (24 × 8 × 6 cm), baited with seeds. The voles were not sedated and were released immediately after instrumentation at the site of capture. They were held in a cotton bag and weighed using a spring scale. Lotek PicoPip VHF collars weighing just under 1.0 g were fitted to six adults, one male and one female in each of the three years 2021–2023. Collar weight was maximum 2.8% of body weight. The transmitter’s battery had a theoretical life length of 38/50 d and the antenna was incorporated into the collar with no extending part. The receiver was FM-100 from Advanced Telemetry Systems, Inc. and the antenna a 4-element Yagi from Televilt International AB. Because this was only a small pilot study with very few animals, the data collected were not suitable for statistical testing.

Voles were tracked by triangulation and homing in, although I tried to keep a minimum distance of 5 m. Positions (fixes) were determined using a handheld Garmin Inc. GPSmap 60Cx. The best accuracy was given as ± 4 m, and positions with an accuracy less than ± 8 m were ignored. Positions were recorded to the nearest meter. Particular attention was given to positions near the boundary of a vole’s known range. A nest site was defined as a specific site that a vole returned to over several days.

Most tracking was performed between 0700 and 2300 h (local summertime), and less during the night. The period of midnight sun extended from the middle of May to 19 July, but bright nights allowed tracking until the middle of August. A fix (position) was generally taken every 30 min during a tracking session. When a vole made more substantial movements, it was followed, and fixes made at 15-minute intervals. This was necessary due to the very low transmitter range. Home ranges were estimated as minimum convex polygons (MCP) in Biotas from Ecological Software Solutions.

Tracking in the three years was spread to cover a wide part of the summer season. Three voles that were tracked over a long time allowed for sub-sampling of the tracking period. At every fix, voles were recorded as either active or passive based on movements or signal variation, or lack of such. The voles were surprisingly little disturbed by my presence, even on occasion running between my legs (this species can be inquisitive and quite bold).

The voles were named after the transmitter’s frequency. The transmitter of vole 023 was retrieved on 27 July 2021 (after expiring), while vole 047 lost her collar. Vole 352 was predated by a stoat and vole 301 disappeared at the same time and site and was probably killed by the same stoat. Vole 980 was predated either by a stoat or a weasel and vole 961 disappeared at the same time and was probably also predated.

Only one other vole species was captured, the red-backed vole *Myodes rutilus*. Its population within the study area was low in all years, as the habitat was not as suitable for this species. The common shrew *Sorex araneus* was frequent, a small hole was cut in the traps to allow shrews to escape [34]. The population size of *C. rufocanus* (and *M. rutilus*) was estimated in the summers of 2017–2023 by live-trapping and mark-recapture. As some *C. rufocanus* were recaptured several times, the range within their trapping sites could be estimated by the MCP method. No adjustments to these ranges were made as they were only meant to give a rough estimate to compare with the ranges of the radio-tracked voles. The area trapped was about 1 ha, where 60–70 Ugglan traps were placed in a grid at 8–15 m intervals. Only voles captured in minimum five different locations were included in the MCP estimates, one individual was caught at 14 locations. Voles were marked by ear-tags or fur-clipping.

## Results

**Table 1:** Home range size for the six *Craseomys rufocanus*, fixes, activity, time period, days tracked and mass

Vole	100%	95%	90%	*N*	Active	Period	Days	Mass
Male 023	36,887	28,339	27,739	471	41.5	22.06–19.07.2021	20	37.0
Male 301	2294	2294	2294	181	45.2	29.05–05.06.2022	8	37.0
Male 980	9673	6013	5815	893	49.5	01.08–06.09.2023	26	43.5
Female 047	2152	1140	1017	131	41.6	22.06–05.07.2021	10	38.5
Female 352	1728	1091	456	176	42.8	29.05–05.06.2022	8	35.0
Female 961	7392	4978	3081	876	50.5	31.07–06.09.2023	27	58.0 P

Home range size in m^2^, 100%, 95% and 90% minimum convex polygons. N = number of fixes. Active = activity in percent of day. Period = time period with collar fitted, Days = number of days tracked. Mass in g when the collar was fitted. P = pregnant

**Table 2:** Home range size and activity for three *Craseomys rufocanus* during three time periods

	Male 023			Male 980		Female 961	
Period	MCP	Active	Period	MCP	Active	MCP	Active
22.06–29.06.2021	21,347	33.9	31.07–12.08.2023	5861	48.5	2328	53.8
04.07–10.07.2021	27,835	49.1	18.08–25.08.2023	7869	53.8	2519	50.0
15.07–19.07.2021	23,136	44.6	30.08–06.09.2023	2473	45.9	6181	44.2

Period = time period tracked. MCP = Home range size in m^2^, 100% minimum convex polygons. Active = Activity in percent of day

**Fig. 2:**
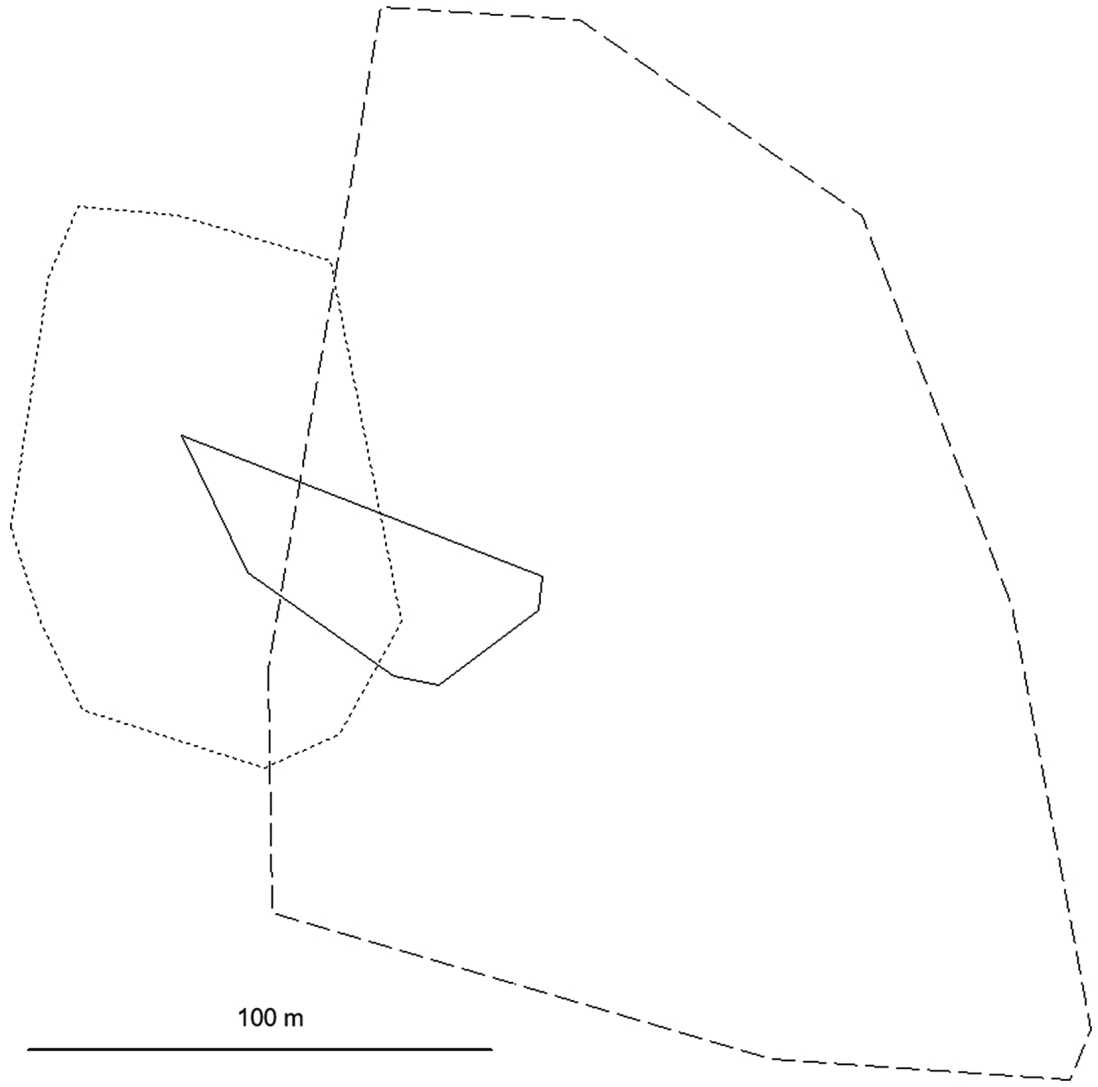
Home ranges (100% MCP) of the three *Craseomys rufocanus* males in three different years; medium line length = male 023, continuous line = male 301, short line length = male 980

**Fig. 3:**
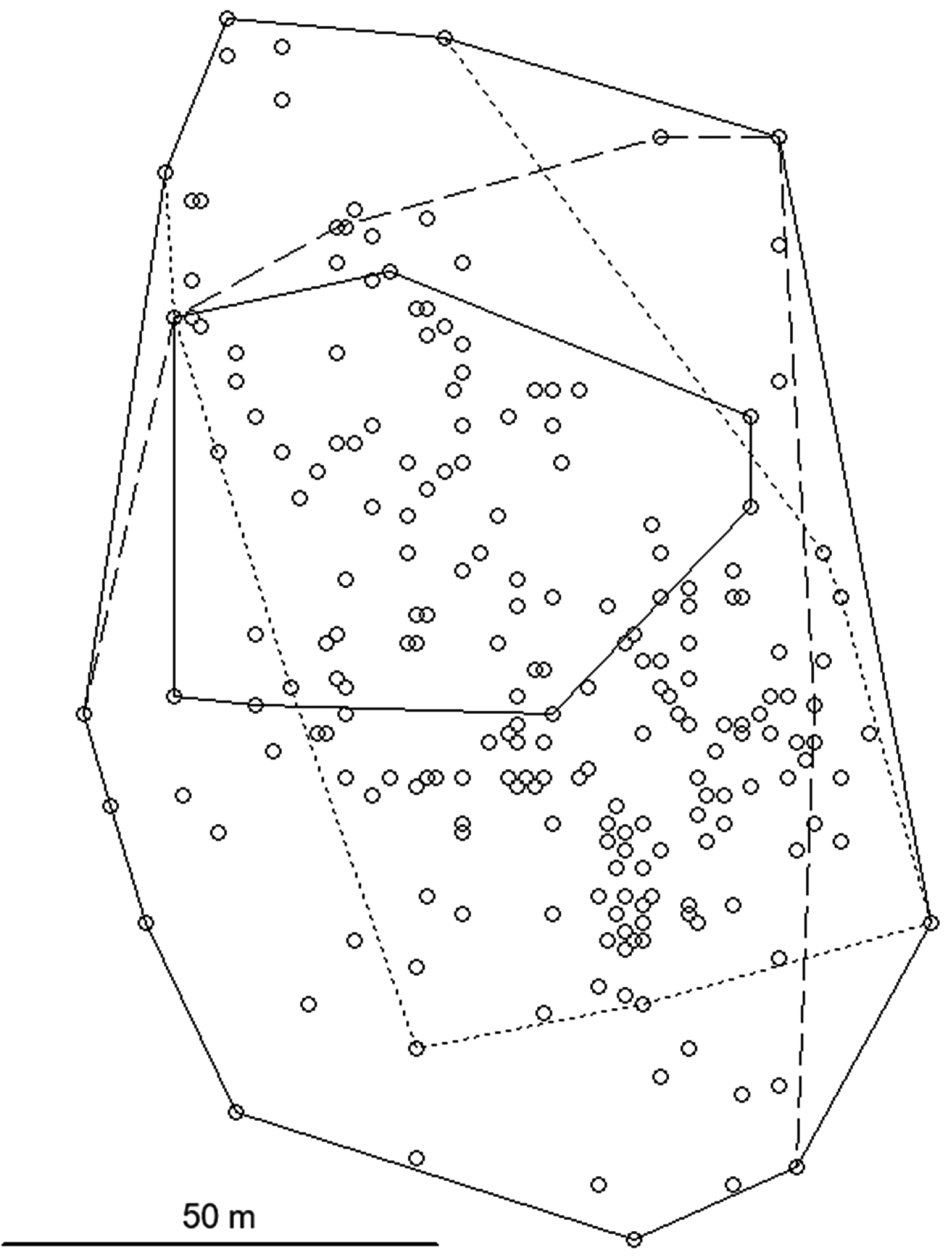
Home range of the *Craseomys rufocanus* male 980 in three periods of the summer 2023; short length lines = period 1, medium length lines = period 2, continuous line = period 3 (see Table 2)

In 2021, the *C. rufocanus* population size in the trapped area was extremely low, in 2022 it was very low, whereas it was high in 2023 with about a ten-fold increase between the last two years. A huge variation in the size of home ranges was found (Table 1). Because of the small number of voles, different time periods and lengths of time during which the voles were tracked, an estimate of a mean male and female range size is unwarranted. Male 023 moved across a particularly large area of 3.60 ha (Table 1; Fig. 2), using a large range in each of the three time periods (Table 2). Male 301 had a small home range (Table 1) despite a small population size. The home range of male 980 was intermediate in size. This male substantially reduced his range in the last time period (Table 2; Fig. 3) when reproduction may have ended, to a size similar to that of male 301.

**Fig. 4:**
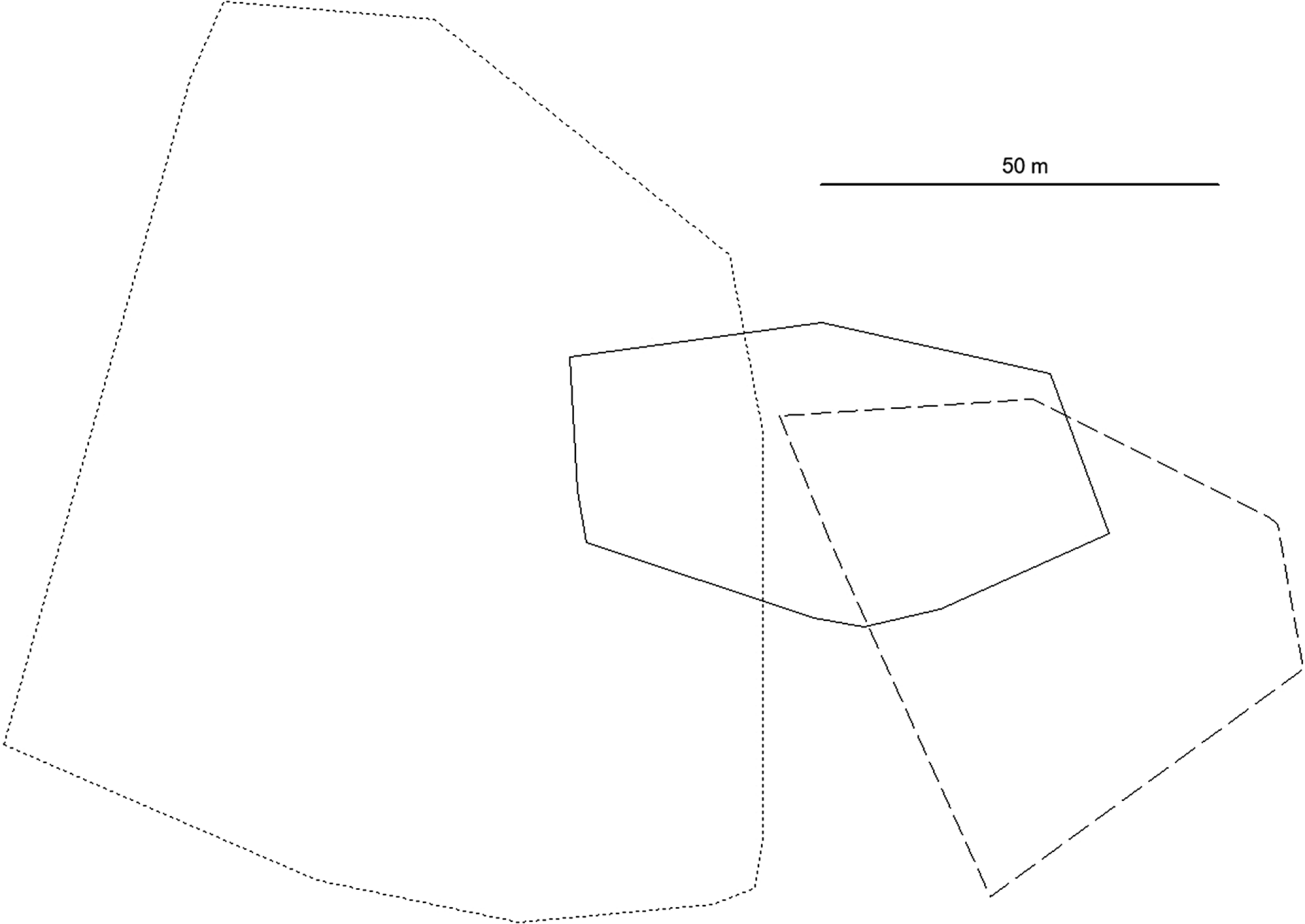
Home ranges (100% MCP) of the three *Craseomys rufocanus* females in three different years; medium line length = female 047, continuous line = female 352, short line length = female 961

**Fig. 5:**
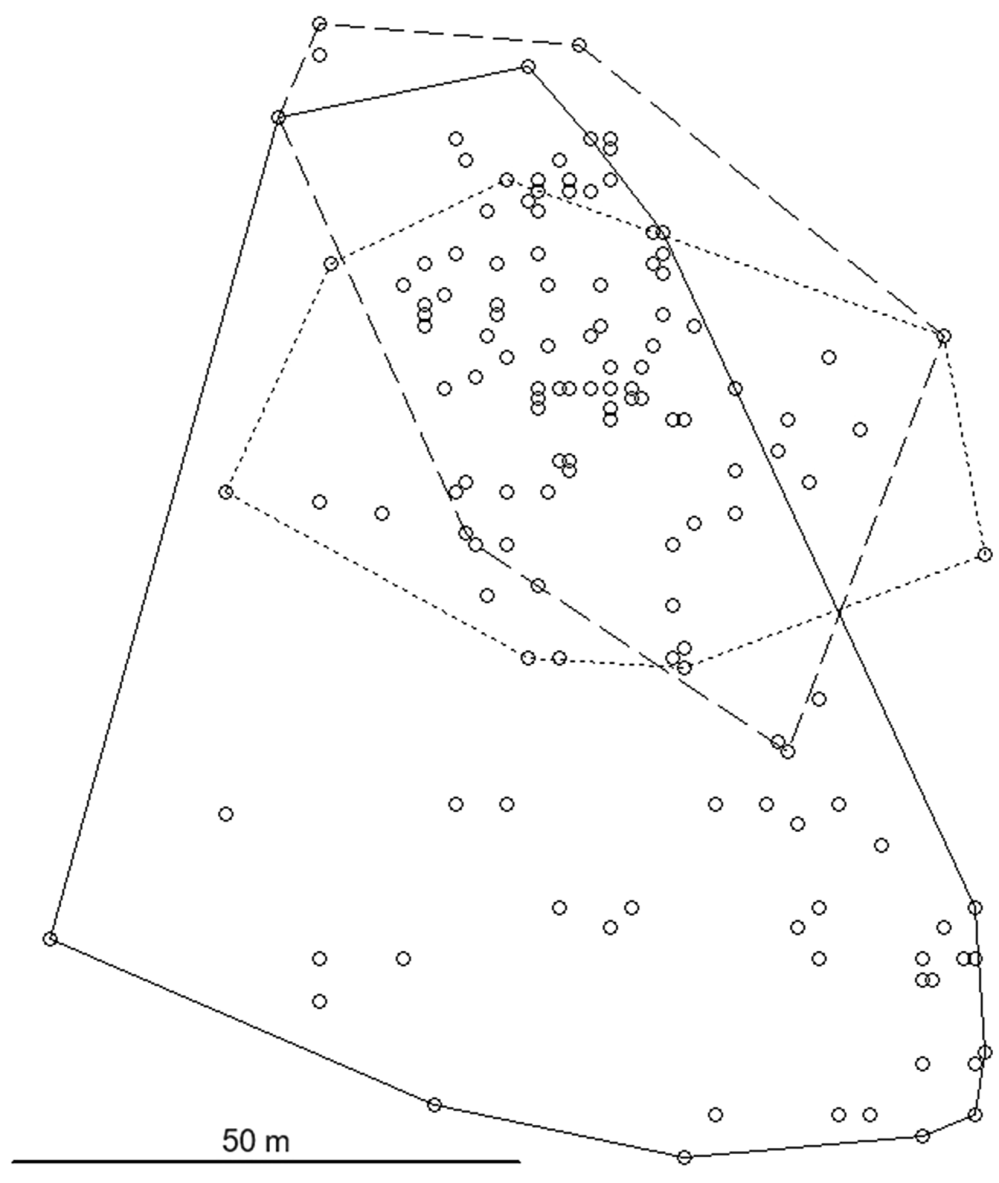
Home range of the *Craseomys rufocanus* female 961 in three periods of the summer 2023; short length lines = period 1, medium length lines = period 2, continuous line = period 3 (see Table 2)

The three females in most of the study periods had ranges around 2 000 m^2^ and usually smaller than those of the males (Table 1; Fig. 4). However, female 961 greatly increased her range towards the end of the study period in 2023 (Table 2; Fig. 5), to the extent that her range was much larger than that of the contemporary male 980. When trapped she was heavily pregnant, with the pups born 1–2 days later. Her ranges in the first two time periods of 2023 were similar (Fig. 5), although she moved about 20 m to a different nest site within her range in the second period, possibly producing a new litter. In the third period she started to move more extensively and used several nest sites (Table 2; Fig. 5), but always returned to her original range. This resulted in the overall large home range size. In total, female 961 used four nest sites. Nest sites of all voles most often were found in a particular structure at the base of birch trees, comprised of live stems and dead stumps of a single tree and accumulated twigs, leaves and debris. Sometimes it was located under rocks. No indication of digging burrows was found.

The males moved much more frequently between sites, but still rested regularly at certain sites. Male 980 used eight nest sites. The home range of male 980 and female 961 included part of a single-lane dirt road. Vegetation encroached both sides of the road that, in the first part of the study, was three m wide. This width increased to 5–6 m following a thorough clearance of the vegetation on both sides. Subtracting the approximate road surface from the home range of male 980 would have decreased his range by about 285/523 m^2^, before and after the vegetation clearance respectively. The reduction would have been a little smaller for female 961. Both tacked voles regularly crossed the road through a culvert (a dry storm drain). When female 961 started to explore a wider area in the last period, she encountered a slightly larger dirt road that was never crossed, perhaps limiting her explorations. Male 023 did cross this road, however, through a culvert on a few occasions.

Although least data were collected in 2022 because both voles were predated only eight days into the study (Table 1), they were still sufficient to estimate the home range sizes for this short period. For male 301 the proportion of the mapped range reached 94.6% at only 88 fixes. For female 352 the proportion reached 99.3% at 155 fixes. A larger number of fixes was needed for voles with larger ranges, e.g., for male 023 the proportion mapped reached 87.6% at 225 fixes.

Despite males roaming more than females, they were not more active (Table 1). The amount of time used in activity was not directly proportional to home range size, except perhaps for male 980 in the three time periods (Table 2). The differences both between and within individuals were quite small (Tables 1 and 2), all being active for slightly less than half the day. Although female 961 increased her range in the third period, she reduced her activity level (Table 2).

The 1 ha trapped area was relatively small compared to a vole home range. Based on CMR the mean home range size for eleven males was 882.1 ± 789.7 m^2^ and for eight females 695.4 ± 533.5 m^2^, the maximum range was 2 145 m^2^ for males and 1 779 m^2^ for females. The ranges found by CMR were correlated with the number of positions recorded (*r* = 0.63, *n* = 19, *p* < 0.01). In any given trapping period, the captures of both males and females appeared to be more clustered in space than randomly dispersed.

## Discussion

Although no statistical test was performed on the present data, the results indicated well which factors may influence the home range size of *Craseomys rufocanus*. In the white-footed mouse *Peromyscus leucopus*: “… home range size and movements of males may depend on the dispersion of females” and “…[males] use a mobile search strategy at low densities when females are widely dispersed, but become territorial and mate polygynously at high densities when females are closely spaced” [8]. For the field vole *Microtus agrestis* it was concluded that home range size was related to density, with larger ranges and greater overlap in males than in females [7, 11]. Male bank voles *Myodes glareolus* showed two different breeding tactics: higher reproductive output related to larger home ranges or lower reproductive output related to smaller home ranges [21]. The home range size and movements of male *C. rufocanus* in this study were related to reproduction and density and primarily to the number of available females [29]. Outside the reproductive season, males used relatively small ranges, similar to those of females. One male in a year of very low density moved across a particularly large area, but apparently found very few females and regularly returned “home”.

During the breeding season, females had much smaller ranges than males, and changing nest site between litters incurred relatively little change in range use by one vole. When reproduction ended this female moved across a larger range, perhaps exploring options elsewhere. Females did not appear to be particularly territorial and, in years of high density, overlap of ranges must have been extensive, contrary to a previous conclusion [31]. In the six voles tracked, home range size was unlikely to have been related to mass, and among all the voles captured no sexual difference in mass was found (but pregnant females would obscure a potentially small difference). Maximum male mass in my study area was 54 g. A large range did not appear to be related to the proportion of activity during the day, as measured by radio-tracking.

Ranges estimated by the CMR method clearly underestimated the actual range of most voles. Larger numbers of recaptures and a larger trapped area would most likely have given more correct range estimates, but this method was generally not reliable as many of these voles extended their range to and possibly beyond the borders of the trapped area. This is contrary to that found in the southern red-backed vole *Myodes gapperi* [18].

## Conclusion

As found in this study, the home range of *C. rufocanus* in the summer was more dynamic and variable than reported in most studies of voles, despite the small sample size. For males the most important factor seems to have been the number of reproductively active females, for females the most important factors could have been food and shelter. Nursing and attending young would require frequent nest visits and limit the females’ range. That males were not more active than females was surprising, indicating that females need more time for feeding and rearing young. The voles were most likely feeding a large part of their active time, and the bursts of running were short even in males. Outside the reproductive season, the voles may reduce their range and possibly aggregate [13, 31, 35], but perhaps temporarily increase their range in search of pastures new in which to overwinter. Hence, male ranges may become similar to those of females and clustering may occur (31, 35, 36). The home range of *C. rufocanus* appears to be very dynamic and shifting according to needs and circumstances. This needs to be considered both in studies of population dynamics and landscape use, including conservation issues.

### Electronic supplementary material

Below is the link to the electronic supplementary material.

Supplementary Material 1

## Data Availability

No datasets were generated or analysed during the current study.
