# Supplementary figures and images for: Dynamic home range of the grey-sided vole Craseomys rufocanus: a pilot study
Source: BMC Zool. 2024 Jul 15;9:16. doi: 10.1186/s40850-024-00209-9 (PMC11247767; doi:10.1186/s40850-024-00209-9)

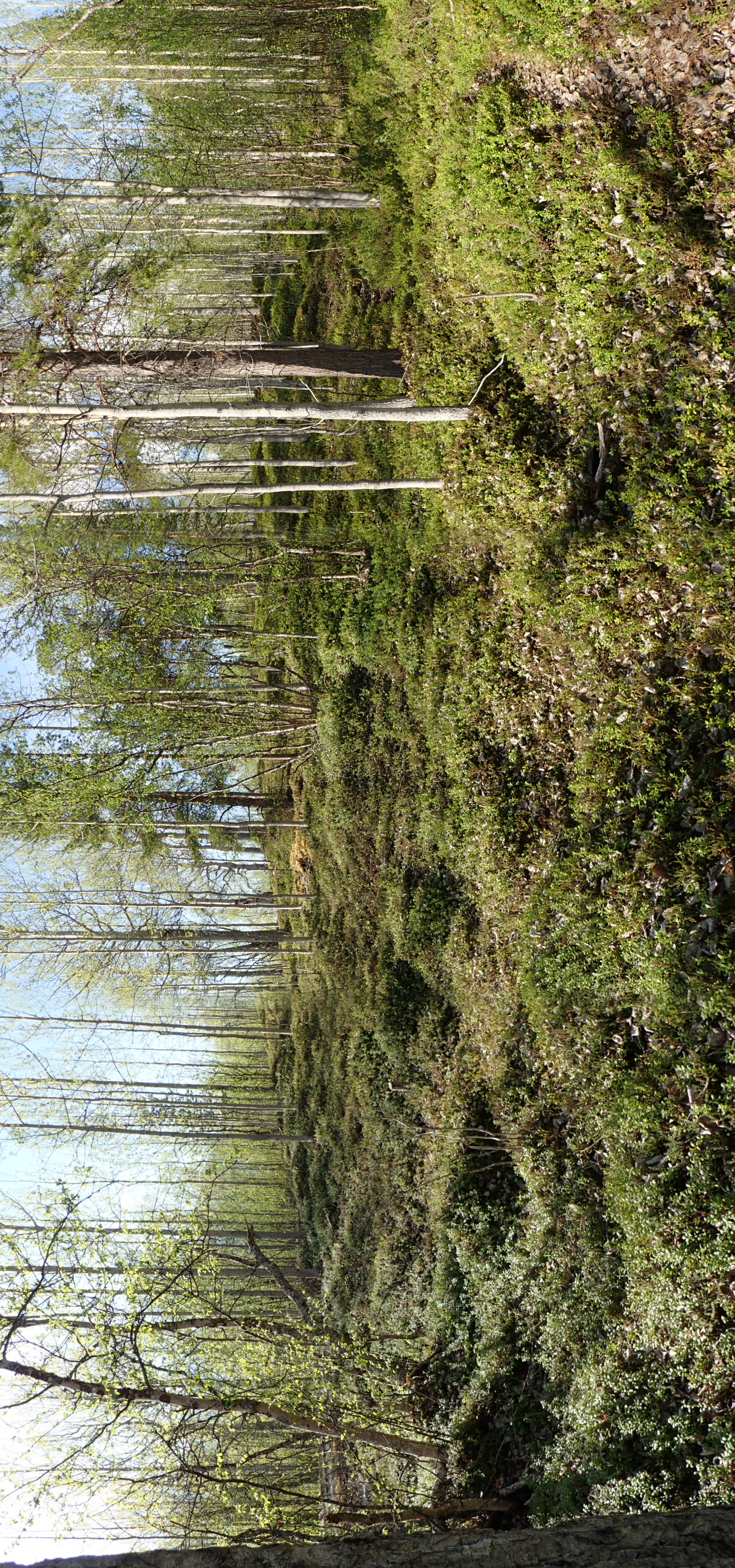

Supplement: Supplementary file 1 — Supplementary Material 1 [file 40850_2024_209_MOESM1_ESM.jpg]
